# MiRNA Deregulation Distinguishes Anaplastic Thyroid Carcinoma (ATC) and Supports Upregulation of Oncogene Expression

**DOI:** 10.3390/cancers13235913

**Published:** 2021-11-24

**Authors:** Danny Misiak, Marcus Bauer, Jana Lange, Jacob Haase, Juliane Braun, Kerstin Lorenz, Claudia Wickenhauser, Stefan Hüttelmaier

**Affiliations:** 1 Institute of Molecular Medicine, Martin Luther University Halle-Wittenberg, 06120 Halle, Germany; danny.misiak@medizin.uni-halle.de (D.M.); jana.lange@medizin.uni-halle.de (J.L.); jhaase2@bwh.harvard.edu (J.H.); juliane.braun@merckgroup.com (J.B.); 2 Institute of Pathology, Martin Luther University Halle-Wittenberg, 06112 Halle, Germany; marcus.bauer@uk-halle.de (M.B.); claudia.wickenhauser@uk-halle.de (C.W.); 3 Division of Endocrinology, Diabetes, and Hypertension, Brigham and Women’s Hospital, Harvard Medical School, Boston, MA 02115, USA; 4 Merck KGaA, 64293 Darmstadt, Germany; 5 Department of Visceral, Vascular, and Endocrine Surgery, Martin Luther University Halle-Wittenberg, 06120 Halle, Germany; kerstin.lorenz@uk-halle.de

**Keywords:** thyroid cancer, ATC, microRNAs, miRNAs, diagnostic marker, microRNA-target interaction

## Abstract

**Simple Summary:**

MicroRNAs are essential regulators of gene expression. Their deregulation is associated with a substantial reorganization of the transcriptome in anaplastic thyroid carcinoma (ATC). Here, we present an integrated, combinatorial approach based on miRNA–mRNA sequencing to unravel miRNA–mRNA networks deregulated in ATC. We identify miRNA–mRNA signatures sharply distinguishing ATC and uncover prime oncogenes upregulated by the downregulation of miRNAs in ATC.

**Abstract:**

Anaplastic thyroid carcinoma (ATC) is the most fatal and rapidly evolving endocrine malignancy invading the head and neck region and accounts for up to 50% of thyroid cancer-associated deaths. Deregulation of the microRNA (miRNA) expression promotes thyroid carcinoma progression by modulating the reorganization of the ATC transcriptome. Here, we applied comparative miRNA–mRNA sequencing on a cohort of 28 thyroid carcinomas to unravel the association of deregulated miRNA and mRNA expression. This identified 85 miRNAs significantly deregulated in ATC. By establishing a new analysis pipeline, we unraveled 85 prime miRNA–mRNA interactions supporting the downregulation of candidate tumor suppressors and the upregulation of bona fide oncogenes such as survivin (BIRC5) in ATC. This miRNA-dependent reprogramming of the ATC transcriptome provided an mRNA signature comprising 65 genes sharply distinguishing ATC from other thyroid carcinomas. The validation of the deregulated protein expression in an independent thyroid carcinoma cohort demonstrates that miRNA-dependent oncogenes comprised in this signature, the transferrin receptor TFRC (CD71) and the E3-ubiquitin ligase DTL, are sharply upregulated in ATC. This upregulation is sufficient to distinguish ATC even from poorly differentiated thyroid carcinomas (PDTC). In sum, these findings provide new diagnostic tools and a robust resource to explore the key miRNA–mRNA regulation underlying the progression of thyroid carcinoma.

## 1. Introduction

Thyroid cancer of follicular origin is the most common endocrine malignancy, with significantly increasing incidence. The majority of these thyroid carcinomas (TC) are further classified into groups of well-differentiated follicular (FTCs; ~10–15% of cases) and papillary (PTCs; ~80% of cases) thyroid carcinomas, poorly differentiated thyroid carcinomas (PDTCs), and the most aggressively undifferentiated, anaplastic thyroid carcinomas (ATCs; ~1–2% of cases) [1,2]. Well-differentiated thyroid carcinomas (WDTCs), including FTCs and PTCs, typically have a good prognosis with a >98% five-year survival. In contrast, ATC presents a nearly uniformly fatal disease. It is highly aggressive (median survival ~4–6 months) and accounts for most thyroid cancer-associated deaths due to a rapidly evolving tumor mass, invasion of the head and neck region and systemic metastases [1,2,3,4].

At the genome level, ATC is characterized by the accumulation of various mutations in BRAF and RAS as well as recurrent mutations in ALK, APC, AXIN1, CTNNB1, PIK3CA, PTEN, TERT, and TP53 [1,5,6,7]. These also occur in WDTCs at variable incidence and frequency [6]. Thus, these somatic mutations largely fail to provide a sufficient basis for the tremendous reorganization of the ATC transcriptome, recently deciphered by the comparative mRNA sequencing of ATC, WDTC (FTC and PTC) as well as nonmalignant thyroid samples [8]. These studies indicated that compared to ATC, WDTCs show a rather moderate change in gene expression in contrast to nonmalignant thyroid carcinomas. The severe reorganization of the transcriptome observed in ATCs results from a multi-layered deregulation at the epigenetic [9], transcriptional, and post-transcriptional levels [6,8]. This was comprehensively emphasized by deciphering the transcriptome landscape of ATCs in comparison to WDTCs [8]. These investigations unraveled that ATCs are distinguished by a strong upregulation of MYC expression and a potent posttranscriptional regulator of mRNA fate, the IGF2 mRNA binding protein (IGF2BP1). The main role of the oncofetal IGF2BP1 is the enhancement of oncogene expression by impairing the miRNA-directed downregulation [10,11,12,13]. This observation suggested that the miRNA-dependent regulation of gene expression substantially underlies the reported reprogramming of the ATC transcriptome. Various studies have described disturbed miRNA expression in ATC and other thyroid malignancies [14,15,16]. This deregulation was further linked to disease-promoting roles such as the enforcement of TGFB/SMAD/ZEB-driven epithelial–mesenchymal transition (EMT). The decreased expression of microRNAs of the miR-30/-200 families in ATC promotes an upregulation of TGFB, SMADs, and ZEBs resulting in a reduced expression of epithelial markers such as e-cadherin (CDH1) [17].

MicroRNAs (miRNAs) are small, noncoding RNAs (ncRNAs) with a length of 19–22 nucleotides. Their general role is the inhibition of gene expression, mainly by targeting the 3′-UTR of mRNAs upon incorporation in the RNA-induced silencing complex (RISC) [18]. MiRNA–RISC–mRNA association results in the shortening of the mRNAs’ poly-A tail leading to an impaired mRNA translation as well as (more prominently) an accelerated decay of the target transcripts. Mostly associated with the only partial complementary of the miRNA and target sequence, miRNAs influence multiple target transcripts in a largely context-dependent manner. Accordingly, they serve multiple roles in essentially all biological processes, and their deregulation has been associated with various diseases, most prominently cancer [19]. Depending on the target spectrum and deregulation of expression observed in cancer, two major classes of miRNAs are distinguished in malignancies. Whereas tumor-suppressive miRNAs, e.g., of the let-7 or miR-200 families, are typically decreased in cancer leading to the upregulation of pro-oncogenic factors, oncomiRs, e.g., miR-21-5p, tend to be upregulated and suppress the expression of tumor-suppressive factors. The role of miRNAs in cancer and thyroid carcinoma progression is emphasized by the mutational burden in the miRNA biogenesis machinery. Recent studies have shown that disturbed miRNA maturation due to mutations in DGCR8, a partner of the core primiRNA processing factor Drosha, is associated with the progression and invasion of PTC and FTC through the deregulation of miRNAs [20]. In addition, mutations in the cytoplasmic RNAse Dicer, which is essential for premiRNA processing and RISC loading, have been associated with cancer progression, including thyroid carcinoma [21,22]. The deregulation of miRNA expression due to disturbed miRNA biogenesis was proposed to underly changes in proliferation, migration, and EMT in thyroid cancer. Consistently, disturbed DICER1 expression and mutations within the RNAse domain were associated with reduced survival probability, probably involving the downregulation of tumor-suppressive miRNAs [22].

Despite various reports on disturbed miRNA expression and regulation in thyroid cancer, the diagnostic and therapeutic potential of miRNAs as well as their role in shaping the ATC transcriptome remain largely elusive. A major limitation of these studies is the lack of comparative analyses addressing miRNA and mRNA expression from identical samples in distinct thyroid carcinomas of follicular origin. Accordingly, the studies presented here provide the first report on miRNA deregulation based on the aforementioned study design. To tackle the substantial amount of data and putative miRNA–mRNA interactions to be considered, we established an analysis pipeline, which aims to evaluate the disease-promoting role of miRNAs in thyroid carcinoma progression. In accordance, we identified cancer hallmarks likely controlled by miRNA deregulation in ATC and demonstrated that the downregulation of miRNAs is associated with a severe upregulation of oncogenes such as survivin (BIRC5). The validation of the two main pro-oncogenic factors, TFRC and DTL, in an independent tumor sample cohort verifies the developed analysis pipeline and confirms a signature comprising 65 genes of primarily miRNA-dependent transcripts distinguishing ATC.

## 2. Materials and Methods

### 2.1. Patient Samples

The presented study comprises 10 ATCs, 6 PTCs, 6 FTCs, and 6 nonmalignant human thyroid (NT, normal thyroid) samples collected from 1999 to 2012 at the Department of Visceral, Vascular, and Endocrine Surgery, University Hospital Halle, Germany. The clinical characteristics and further sample information were previously reported [8].

### 2.2. Small RNA Library Construction, High-Throughput Sequencing, and Differential Expression Analysis

The total RNA of patient samples was extracted from primary tumor tissue using the Qiagen ALLprep tumor protocol with miRNeasy kits (Qiagen). A total of 500 ng of total RNA was used for the small RNA library preparation with the TruSeq™ Small RNA sample prepkit v2 (Illumina) according to the instructions of the manufacturer. The barcoded libraries were size restricted from 140 to 165 bp. A pool of 10 libraries was used for cluster generation at a concentration of 10 nM using an Illumina cBot. Sequencing of 51 bp was performed with an IlluminaHighScan-SQ sequencer at the sequencing core facility of the IZKF Leipzig (Faculty of Medicine, University Leipzig) using version 3 chemistry and flowcell according to the instructions of the manufacturer. Demultiplexing of raw reads, adapter trimming, and quality filtering were done according to Stokowy et al. [23]. Unstranded single-end reads with 51 bp in length were trimmed for adapter and low-quality sequences using Cutadapt (v 1.18). Trimmed reads were mapped to the human genome (hg38 UCSC) using Bowtie2 (v 2.2.6, [24]), allowing for one mismatch in the seed region (parameter-N 1). Subsequent, mapped reads were summarized using featureCounts (v 1.6.3, [25]) and miRBase (v 22, [26]) annotations.

Differential expression of miRNAs was determined using R package edgeR (v 3.28, [27]) utilizing the trimmed mean of M-values (TMM, [28]) normalization of read counts to reduce bias by variable overall miRNA abundance. Normalized log2 CPM (counts per million mapped reads) values of miRNA expression were used (Table S5). Differential expression of mRNAs, as recently reported [8], was reanalyzed according to the same procedure with an updated human genome version (from GRCh19 to 38, Table S1). Further information for RNA-seq tumor samples can be retrieved from [8] and are available from GEO accession GSE126698 (https://www.ncbi.nlm.nih.gov/geo/query/acc.cgi?acc=GSE126698, accessed on 19 November 2021).

### 2.3. Unsupervised Expression Analysis

CPM values from small RNA-seq expression data were log2 transformed, and low abundant miRNAs with average CPM < 100 were filtered out. A principal component analysis (PCA) was performed with R package PCAtools (v 2.4.0, https://github.com/kevinblighe/PCAtools, accessed on 19 November 2021). A hierarchical cluster analysis was performed by using the R package gplots/heatmap.2 (v 3.1.1, https://github.com/talgalili/gplots, accessed on 19 November 2021). Expression values were scaled for each miRNA to compare different miRNAs from low to high expression, which is equivalent to the range (−1, 1) of scaled log2 CPM values.

### 2.4. Prediction of microRNA-Target Interactions and Binding Site Positions

Predicted and experimentally validated miRNA–mRNA interactions were retrieved from miRWalk (v 3, [29]). A default binding probability of at least 95% (score ≥ 0.95) was used as a binding prediction threshold within the 3’-UTR binding site position of the putative miRNA target genes. In addition, information of the two prediction tools TargteScan and miRDB [30,31], provided by miRWalk, was considered.

Further, the exact positions of the predicted miRNA–mRNA binding sites by miRWalk were considered. More stringent thresholds were applied, presuming a binding probability of 100% at the 3’-UTR region, and an average expression of the targeting miRNA with CPM > 100 across all samples was analyzed. To account for different transcript variants and therefore different 3’-UTR regions, a meta 3’-UTR region was constructed by using the R package Gviz (v 1.36.2, [32]). Since miRWalk only provides miRNA binding site information based on transcript positions of the corresponding 3’-UTR, the locations were recalculated by applying the transcriptToGenome method from the R package ensembldb (v 2.16.4, [33]). Finally, only miRNAs targeting the determined target mRNAs were considered that show a binding site within the meta 3’-UTR and therefore target at least one mRNA transcript.

### 2.5. Pan-Cancer Loss of Function Studies

To integrate essentiality information of the determined miRNA target genes in ATC, pancancer loss-of-function CRISPR screens of nine ATC-derived cell lines (8305C, 8505C, MB1, ASH3, BHT101, CAL62, HOTHC, HTCC3, KMHDASH2) were utilized for a dependency analysis, using the Broad Institute Cancer Dependency Map (DepMap) portal (v 21Q1, [34]). Subsequently, average dependencies were calculated for identified target genes across all cell lines (Table S2).

### 2.6. TCGA Based Survival Analysis

The Cancer Genome Atlas (TCGA) was used to investigate target potential on survival and prognosis based on public data. Notably, comprehensive clinical data in combination with RNA-seq expression data is only available for 502 WDTC samples and does not include any ATC. Irrespective of this shortcoming, this approach was chosen, since clinical data on the tumor samples analyzed in this study remained partially incomplete, and the number of samples was too small for proper data evaluation. Nevertheless, the obtained survival rates were integrated in order to determine a tendency in the ATC based on the expression of the examined miRNA target genes in the PTC. Target mRNAs of selected miRNAs (cf. 2.7) were filtered for expression (≥1 FPKM in total) across all samples, log2 transformed, and associated with available clinical data from the TCGA cohort. A log-rank test was performed with the R package survival (version 3.2-11, https://CRAN.R-project.org/package=survival, accessed on 19 November 2021). High and low expression groups were separated by the respective median of the log2 transformed normalized expression values to investigate the association of altered mRNA expression and survival probability. Finally, hazardous ratios (HR) were determined by differences of the Kaplan–Meier survival curves for both respective groups by the log-rank test (Table S3). We expected that oncogenic factors controlled by miRNA downregulation to be characterized by hazard ratios (HR) greater than 1. In contrast, HR values smaller than 1 were considered indicative for a rather tumor-suppressive role of factors decreased by upregulated miRNAs.

### 2.7. MicroRNA-Target Selection, Interaction Score, and OncoScore Calculation

MiRNAs deregulated in ATC were selected by significantly (FDR < 0.01) deregulated expression in ATC compared to all other (WDTC and NT) samples analyzed. In addition, only miRNAs with an average expression of 100 CPM (count per million) across all samples were considered. To identify prime candidate target mRNAs of differentially expressed (DE) miRNAs, we established a scoring system allowing the evaluation of miRNA–mRNA interactions by one interaction score (IS). The IS was determined based on the following considerations and scoring:

(1) Initially candidate miRNA–mRNA interactions were determined via miRWalk. Only interactions with a binding score > 0.95 were considered further and scored with the respective binding probability value determined by miRWalk (0.95–1). In addition, the binding predicted by miRDB and TargetScan were scored with 1 or 0 (no binding predicted), respectively. The sum of the binding scores was divided by three to yield a maximum score of 1 for the most likely (predicted with high probability by all three databases) candidate miRNA–mRNA interactions.
(2) The scaled fold change (logFC) of the target mRNA expression in ATC was considered with maximum upregulation = 1 and downregulation = −1, respectively.
(3) In addition, the fold change of mRNA was further scored with 1 if (a) the fold change of target mRNA expression showed inverse association with miRNA deregulation, and (b) mRNA abundance was significantly changed (FDR < 0.05).
(4) The average essentiality score (ES) determined for genes in ATC-derived cell lines—available via DepMap (cf. 2.5)—was scaled from −1 to 0 (ES < 0) or 0 to 1 (ES > 0) and considered as outlined in the formulas below. This scaling settled on the assumptions that the upregulation of target mRNAs by DN-miRNAs promotes the abundance of factors with a negative essentially score (ES), indicative of a rather pro-oncogenic role. The opposite, downregulation of target mRNAs by UP-miRNAs was expected for factors with a positive ES, indicative of a rather tumor-suppressive role.
(5) The hazardous ratio (HR) of candidate target mRNA expression (cf. 2.6) was determined in the THCA cohort provided by the TCGA and considered as outlined below. HR values were scaled from −1 to 0 (HR < 1) or 0 to 1 (HR > 1).

The IS was determined for UP-miRNAs (1) and DN-miRNAs (2) in ATC on the assumption that upregulation of miRNAs (UP-miRNAs) indicates candidate oncomiRs controlling the expression of rather tumor-suppressive factors. The opposite was expected for downregulated miRNAs (DN-miRNAs), which were expected to contribute to ATC progression by downregulating rather pro-oncogenic factors.

**UP-miRNAs:** IS = (1) − (2) + (3) + (4) − (5) (1)

**DN-miRNAs:** IS = (1) + (2) + (3) − (4) + (5) (2)

Finally, the IS was scaled to a range of [−1, 1] within all interactions of each miRNA separately, reflecting the interaction score of all determined miRNA target interactions for a selected miRNA. The IS can be interpreted as a key value allowing the assessment of individual miRNA–mRNA interactions in respect to (a) probability of relevant regulation (miRNA-directed downregulation of the target mRNAs) and (b) disease relevance of the regulation (e.g., upregulation of oncogenes due to miRNA downregulation). Due to partially distinct formulas for UP- vs. DN-miRNAs, the IS allowed scaling between −1 (unlikely) and 1 (most likely) miRNA-mRNA interactions.

Aiming to allow for an assessment of the most likely miRNA-controlled mRNAs in respect to pro-oncogenic vs. rather tumor-suppressive roles of the respective proteins, we established an *OncoScore* (OS). The OS evaluated how strongly miRNA downregulation promoted the expression of genes with negative essentiality scores determined in ATC-derived cells and to which extend upregulated mRNA expression is associated with reduced survival probability. The opposite was expected for upregulated miRNAs, expected to primarily direct the downregulation of tumor-suppressive factors. Accordingly, the OS considered the above listed criteria (2), (4), and (5) according to the formula: OS = (2) − (4) + (5). Finally, OS values were scaled to [−1, 1].

### 2.8. Gene Set Enrichment Analysis of miRNA Target mRNAs

Gene Set Enrichment Analysis (GSEA) was performed using the R package clusterProfiler (v 4.0.5, [35]) and MSigDB (v 7.4, [36]) gene sets utilizing the Fgsea algorithm and setting the exponent parameter to 0 for the unweighted analysis of pre-ranked expression data. The hallmark gene set collection [36] was applied with a minimum set size of 10 and no upper size restriction (Table S4). Investigated target mRNAs were ranked according to their log2 fold change of mRNA expression in ATC vs. other. To simplify the evaluation of these analyses, we focused on the inhibitory role of miRNAs on mRNA expression suggesting inverse trends of mRNA and miRNA abundance. This implied positive NES (normalized enrichment scores) for gene sets determined for target mRNAs of downregulated miRNAs versus negative NES for target mRNAs of upregulated miRNAs.

### 2.9. Tissue Microarray and Immunohistochemistry

The inhouse tissue microarray (TMA) contains 151 primary thyroid cancer samples (20 ATC, 18 PDTC, 84 PTC, and 29 FTC) collected at the Institute of Pathology between 2014 and 2019 and previously described [8].

Immunohistochemistry was performed on 3 µm thick, consecutive sections of formalin-fixed, paraffin-embedded samples with the Bond Polymer Refine Detection Kit (Leica, DS9800), according to the manufacturer’s instructions on a fully automated immunohistochemistry stainer (Leica Bond). Sections were imaged with an Olympus BX50/51 microscope. Two pathologists (CW and MB) independently scored all samples by using a histoscore (H-score). In brief, the relative amount of tumor cells being positively stained (%) was multiplied by their intensity from 0 (negative), 1 (weak), 2 (moderate), to 3 (intense). Overall expression was classified into absent (0), low (1–100), intermediate (101–200), or strong (201–300). The antibodies anti-TFRC (HPA028598) and anti-DTL (HPA028016) were used from Sigma-Aldrich according to the manufacturer’s instructions. Bone marrow with reactive changes was used as a positive control for all primary antibodies. A negative control without primary antibodies was carried out.

## 3. Results

### 3.1. SmallRNA Sequencing Unravels miRNA Signatures Distinguishing ATC

**Figure 1:**
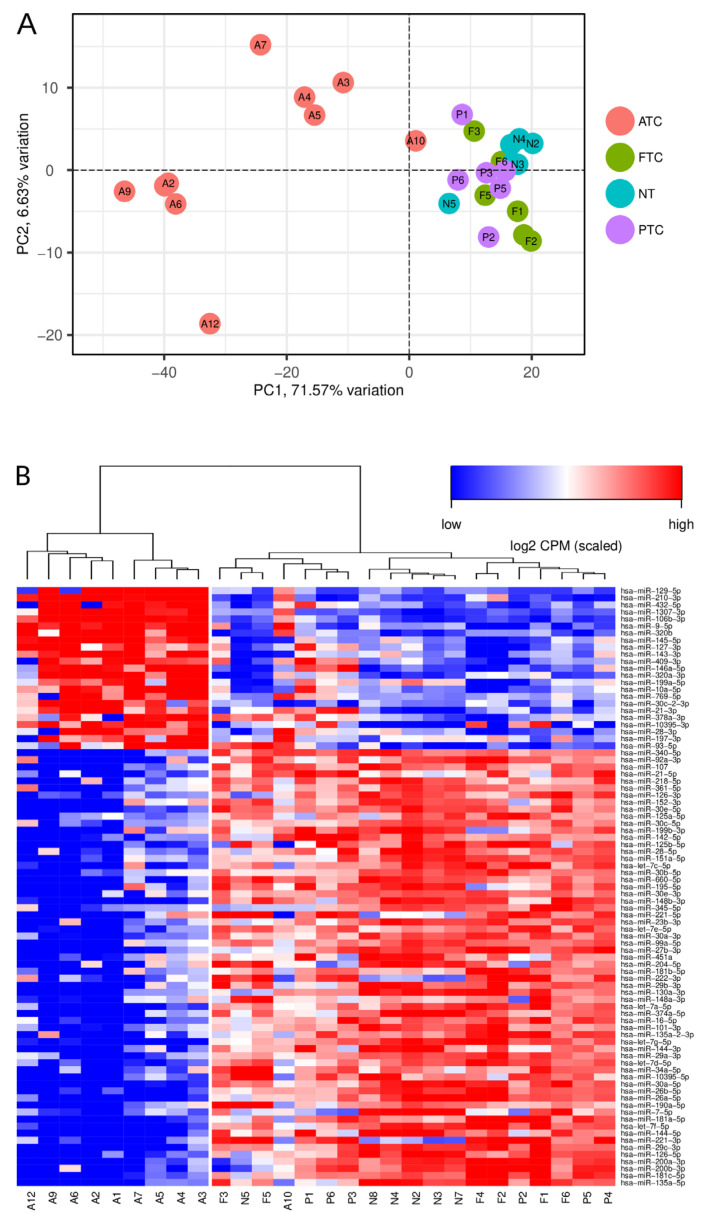
Unsupervised analyses of small RNA expression data from 10 ATCs, 6 PTCs, 6 FTCs, and 6 nonmalignant human thyroid (NT, normal thyroid) samples of 85 miRNAs with significantly deregulated expression (DE) in ATC. (**A**) Principal component analysis (PCA) shows that DE miRNAs distinguish ATC from all other samples analyzed, except for ATC#10. (**B**) Hierarchical clustering of the 85 significantly deregulated miRNAs supports the PCA analyses and sharply distinguishes all ATC samples, except ATC#10. Rows are ordered by average log2 fold change of miRNA expression in ATC samples compared to all other samples analyzed. Normalized log2 CPM (counts per million mapped reads) expression values of identified miRNAs were used for both analyses.

The comparison of miRNAs in ATC compared to all other WDTC and NT samples, which are collectively referred to as “others”, revealed an ATC-specific miRNA deregulation. This resulted in 85 miRNAs with a significantly different expression in ATC and comprised 23 UP-miRNAs and 62 DN-miRNAs (Table S6). A principal component analysis (PCA) of miRNA abundance demonstrated that miRNA expression sharply distinguishes ATC, despite substantial inter-sample variability within the analyzed ATC samples (Figure 1A). This was further evaluated by unsupervised clustering (USC) using expression data derived for the 85 differentially expressed (DE) miRNAs. In support of PCA, USC clearly separated 9 of 10 ATCs from all other samples investigated (Figure 1B). Only one ATC sample (#10) showed an untypical distribution of miRNA abundance, potentially indicating lower tumor content, remaining PDTC/WDTC contamination, or disturbed sample quality. A potential misdiagnosis was excluded based on an independent assessment of samples by pathologists and an mRNA expression by RNA-sequencing (RNA-seq) data derived from the identical samples as well as an immunohistochemical re-evaluation, as described previously [8].

The inspection of substantially deregulated miRNAs overall confirmed prior studies on miRNA deregulation in ATC [14,37,38], e.g., the downregulation of the rather tumor-suppressive let-7 miRNA family, the TP53-driven miR-34c, and the pro-epithelial miRNAs of the miR-200 and miR-30 families [14]. Despite the previously demonstrated upregulation of miRNAs, suggesting oncogenic functions in ATC (oncomiRs), such as miR-146a-5p, we surprisingly observed a significantly decreased expression of the most powerful oncomiR, miR-21-5p, although this miRNA remained among the most abundant in ATC.

### 3.2. MiRNA Deregulation in ATC Guides Gene Expression Shifts Distinguishing ATC

**Figure 2:**
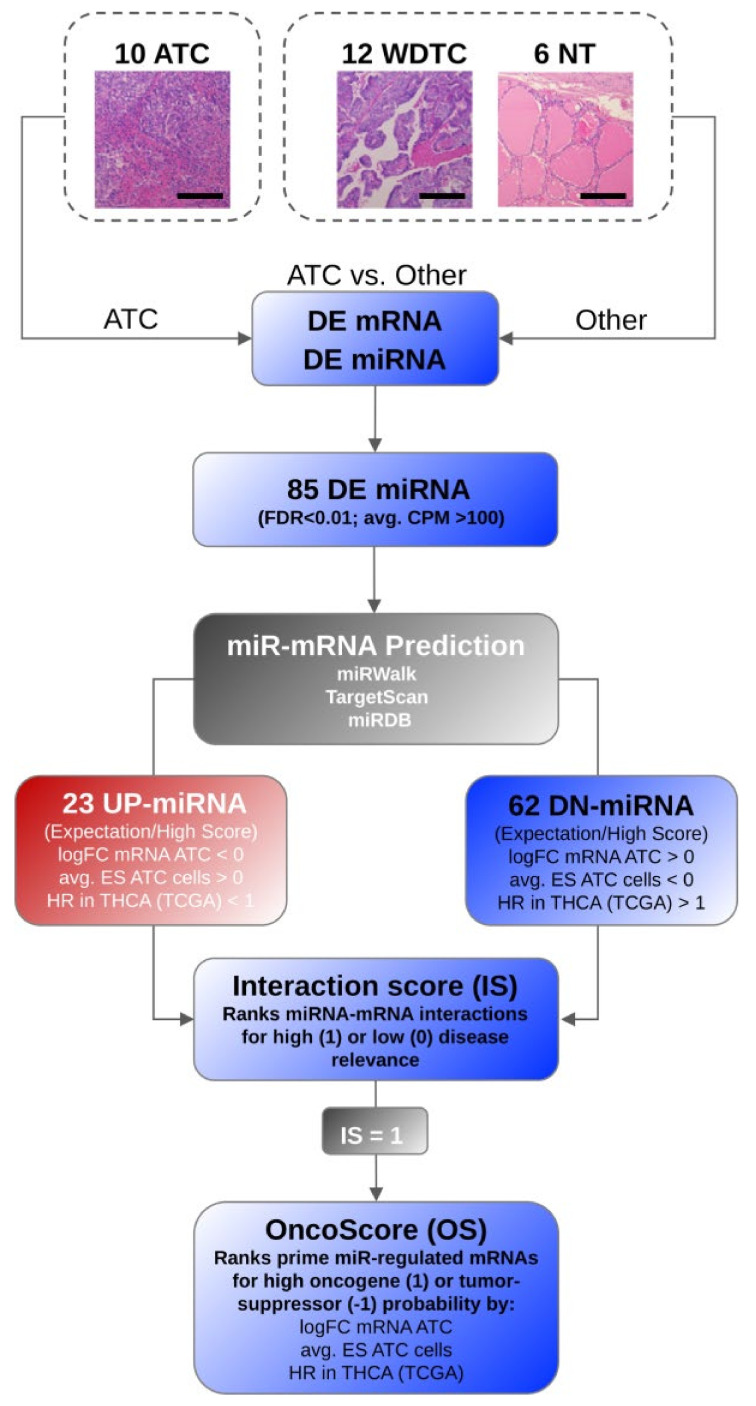
The established analysis pipeline showing the rational for selection of prime miRNA–mRNA interactions in ATC. In brief, the miRNA and mRNA expression were determined by RNA-seq in indicated samples. Differential miRNA expression identified 85 differentially expressed (DE) miRNAs at the threshold of FDR < 0.01 and an average expression > 100 CPM. To identify key effector mRNAs of DE miRNAs, miRNA targeting was analyzed by in silico prediction using indicated databases. Candidate miRNA–mRNA interactions were further ranked by the fold change (FC) of target mRNA expression, average essentially scores (ES) were determined in ATC derived cells for target genes, and hazard ratios (HR) were determined by target mRNA expression in the TCGA thyroid cancer cohort (THCA). This led to an interaction score (IS) for each miRNA-mRNA interaction scaled as indicated. Finally, prime candidate oncogenic and tumor-suppressive target mRNAs were determined for the top scoring (IS = 1) target mRNA of each DE miRNA by the indicated parameters. This allowed a ranking of miRNA-controlled candidate oncogenic and tumor-suppressive factors via a scaled OncoScore (OS), as indicated. Bars indicate a scale of 100 µm.

The deregulation of miRNA synthesis distinguishing ATC suggested that miRNAs significantly contribute to the recently reported reprogramming of gene expression in ATC. Therefore, we established an analysis pipeline to identify candidate key gene sets as well as individual candidate oncogenic and tumor-suppressive factors, showing a deregulation in ATC due to divergent miRNA expression (Figure 2). This was essentially based on the experimentally validated deregulation of miRNA and mRNA expression, as determined by miRNA- and mRNA-seq (cf. [8]) data from identical samples. In brief, candidate target mRNAs (expressed in analyzed samples) of the individual 85 miRNAs deregulated in ATC were predicted. This resulted in 16,073 candidate target mRNAs and a large number of 128,262 distinct candidate miRNA–target interactions. To strengthen the prediction of miRNA–mRNA interactions, additional binding information from two independent prediction tools was included. Moreover, to allow for an evaluation of potentially disease-relevant miRNA–mRNA interactions, the differential expression of mRNAs in ATC was considered. To this end, analyses were based on the major role of miRNAs in reducing target mRNA abundance. Accordingly, miRNA–mRNA interactions were evaluated by the fold change of target mRNA expression providing positive scores for target mRNA upregulation in the case of DN-miRNAs and target mRNA downregulation for UP-miRNAs. In addition, miRNA–mRNA interactions were ranked according to their average essentiality scores determined for the respective genes in ATC-derived cells. Finally, we considered the prognostic value of each candidate target mRNA derived from the TCGA thyroid cancer cohort (THCA; Table S3).

The established pipeline evaluated 14,094 candidate target mRNAs of the 85 miRNAs deregulated in ATC, requiring the investigation of 115,721 miRNA target interactions. This allowed for a comprehensive evaluation of the aforementioned hypotheses to finally derive a scaled (−1/1) interaction score (IS) for each target mRNA association of the 85 deregulated miRNAs (Table S2). From these analyses, we could already derive prior reported miRNA–mRNA regulations as well as novel candidate miRNA target associations with expected roles in thyroid cancer progression.

**Figure 3:**
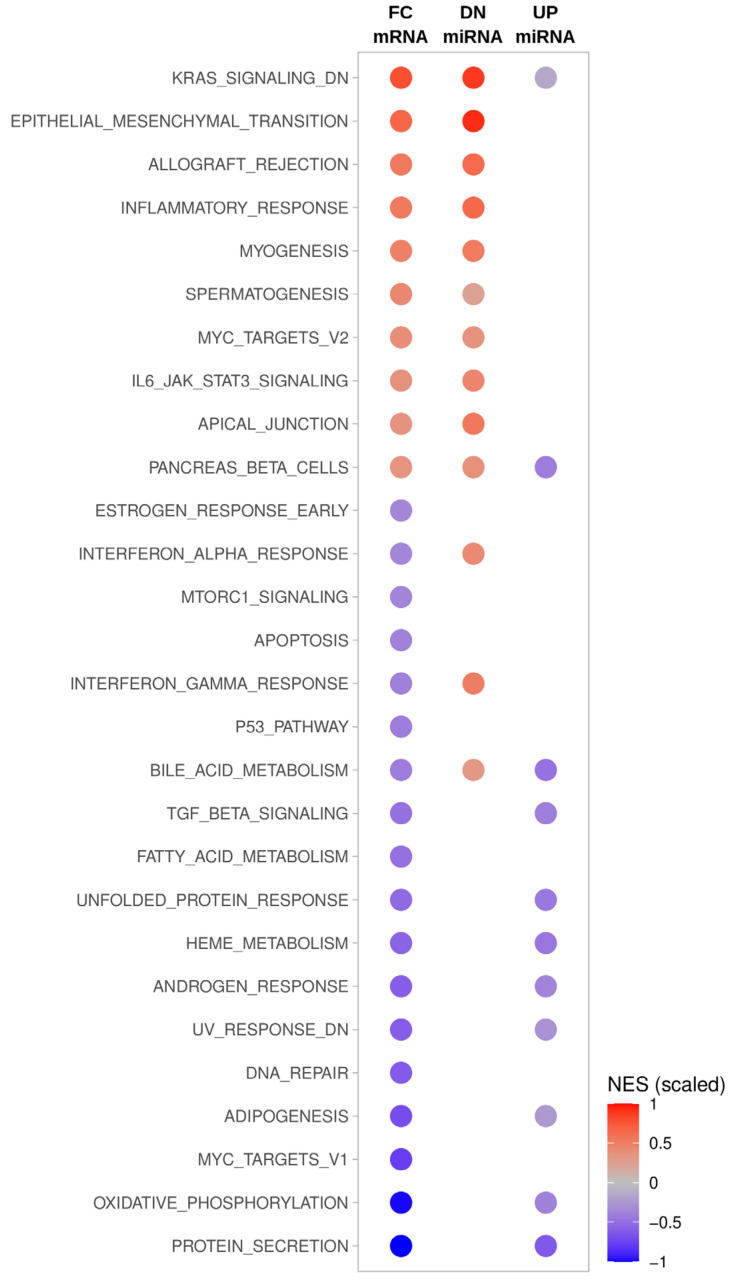
Gene Set Enrichment Analysis (GSEA) of total and miRNA-dependent differential mRNA expression in ATC. Columns indicate GSEA results using distinct mRNA selection: FC mRNA, all expressed mRNAs were ranked by their log fold change of expression in ATC compared to all other samples; DN miRNA, candidate target mRNAs with positive interaction scores (IS) determined for DN-miRNAs were ranked by their log fold change of expression in ATC compared to all other samples; UP miRNA candidate target mRNAs with positive interaction scores (IS) determined for UP-miRNAs also ranked by their log fold change of expression in ATC. Color coding indicates scaled normalized enrichment scores (NES) determined for indicated hallmark gene sets. NES scale bar is indicated.

To evaluate if a miRNA-directed regulation of mRNAs ranked by IS scoring indicates regulation of underlying thyroid carcinoma progression, we performed a Gene Set Enrichment Analysis (GSEA). To this end, target mRNAs of the 85 deregulated miRNAs with positive IS values, indicative of potentially diseases relevant miRNA-mRNA regulation, were considered. All findings for the two sets of deregulated miRNAs, DN- vs. UP-miRNAs, were compared to significantly changed (FDR < 0.05) gene sets identified by GSEA based on an altered mRNA expression in ATC compared to all other samples, as prior described (Figure 3; [8]). A comprehensive presentation of GSEA results is presented in supplementary Figure S1.

An inspection of the GSEA results revealed that the deregulation of specific gene sets and thus of tumor properties is likely driven by miRNA deregulation (Table S4). For instance, the downregulation of miRNAs appears to serve essential roles in supporting the prior described epithelial–mesenchymal–transition (EMT), characteristic for ATC. This supports previous findings indicating that downregulation of the miR-30/200 families promotes TGFB/SMAD-dependent EMT in ATC [17]. On the contrary, a direct link of miRNA deregulation with gene sets downregulated in ATC remains rather vague due to the lack of prior described miRNA–mRNA regulation. However, GSEA strongly suggests that hallmarks of ATC, a proglycolytic shift and elevated hypoxia/anoxia, are promoted by the miRNA-dependent downregulation of factors involved in oxidative phosphorylation.

### 3.3. Key Effectors of miRNA Deregulation in ATC

Since the previous analyses could not provide insights on key effectors of miRNA deregulation in the progression of thyroid carcinoma, we aimed to identify candidate oncogenic and tumor-suppressive factors controlled by miRNA deregulation in ATC. Therefore, we investigated the top candidate target mRNAs derived by IS scoring for each of the 85 deregulated miRNAs (cf. Figure 2). Strikingly, this already revealed mRNAs with an elevated probability of miRNA-dependent regulation, since 12 mRNAs were scored with an IS value of 1 for more than one of the 85 deregulated miRNAs. For three target mRNAs (BIRC5, IGF2BP1, and RRM2), a maximum of four prime-targeting miRNAs were identified that were all downregulated in ATC. This supported prior findings demonstrating, for instance, that the downregulation of the let-7 miRNA family promotes the upregulation of IGF2BP1 and RRM2 in cancer [11,39,40,41].

**Figure 4:**
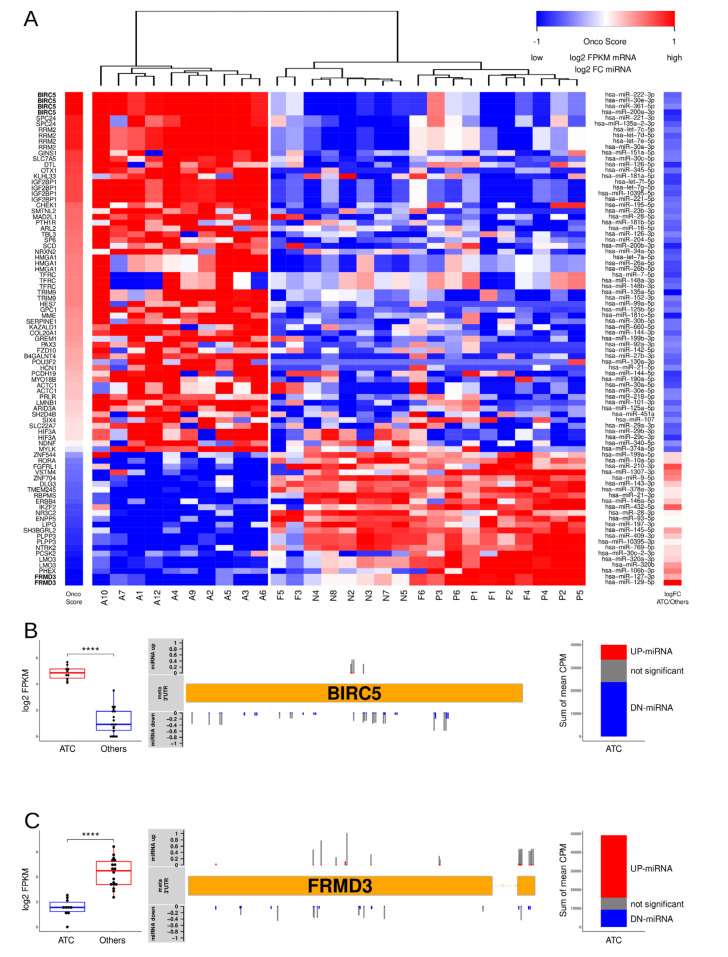
High ranking miRNA–mRNA interactions identify miRNA-dependent regulation of oncogenic and tumor-suppressive factors in ATC. (**A**) Hierarchical clustering of the top scoring (IS = 1) target mRNAs of the 85 DE miRNAs sharply distinguish all ATC samples. Samples were ordered by the scaled OncoScore (OS), indicating the oncogenic (top score 1) vs. tumor-suppressive (top score −1) potential of the respective genes. Gene symbols are indicated in the left panel. Log2 transformed FPKM expression values of indicated mRNAs scaled to low and high expression range [−1, 1] are depicted by a heatmap in the middle panel. Prime targeting miRNAs of indicated mRNAs are indicated in the right panel along with the scaled [−1, 1] fold change of miRNA expression in ATC vs. others. (**B**,**C**) Expression and miRNA targeting of the prime oncogene upregulated by miRNA downregulation (BIRC5, (**B**)) and the prime tumor-suppressor candidate decreased by miRNA upregulation (FRMD3, (**C**)). Boxplots (left panels) show BIRC5 and FRMD3 mRNA (log2 FPKM) expression in ATC and others; **** *p* < 0.0001. Schemes (middle panels) depict the meta 3′-UTR of selected genes with predicted binding sites for targeting miRNAs with average expression >100 CPM. Fold change of miRNA expression is indicated by up/down bars scaled by fold change of expression. Color coding indicates significant deregulation (FDR < 0.01). Nonsignificant in grey. The sum CPM of targeting miRNAs sorted by significant (color coded) or insignificant change of expression (grey) is indicated in the right panels.

To further evaluate the potential impact of the identified 85 key miRNA–mRNA regulations on disease progression, each of the interactions was evaluated by an *OncoScore* (OS), ranking the determined target mRNAs (Figure 4A; Table S7). The potential oncogenic (maximum OS of 1) or tumor-suppressive role (minimum OS of −1) was evaluated by the scaling of the parameters mentioned before. Unsupervised clustering of the respective mRNAs based on their abundance in the 28 analyzed samples strikingly distinguished ATCs from all other samples (Figure 4A). Notably, this also included the prior misclassified ATC sample #10, suggesting that the miRNA-dependent regulation of mRNAs is a suitable diagnostic tool for identifying ATC based on transcriptome data. Moreover, the OS identified BIRC5 (survivin) as the top scoring oncogene controlled by the deregulation of miRNAs in ATC, specifically the downregulation of miR-222-3p, miR-30e-3p, miR-361-5p, and miR-200a-3p.

Notably, although an inhibitory role on BIRC5 was proposed for all four of these miRNAs, only miR-222-3p is considered for survivin-focused replacement strategies in cancer treatment [42]. On the other side of the spectrum, the most prominent miRNA-dependent regulation of a barely investigated candidate tumor suppressor [43], FRMD3, is associated with the upregulation of miR-127-3p and miR-129-5p. The further evaluation of these two prime candidate effectors of deregulated miRNA expression in ATC confirmed a striking upregulation of survivin expression in ATC (Figure 4B). The opposite was observed for the FRMD3 mRNA, which is substantially downregulated in ATC (Figure 4C). Most notably, even when considering all candidate miRNA–mRNA interactions, we observed an overall trend of downregulation for miRNAs controlling survivin (BIRC5), whereas candidate targeting miRNAs were rather upregulated in the case of FRMD3.

**Figure 5:**
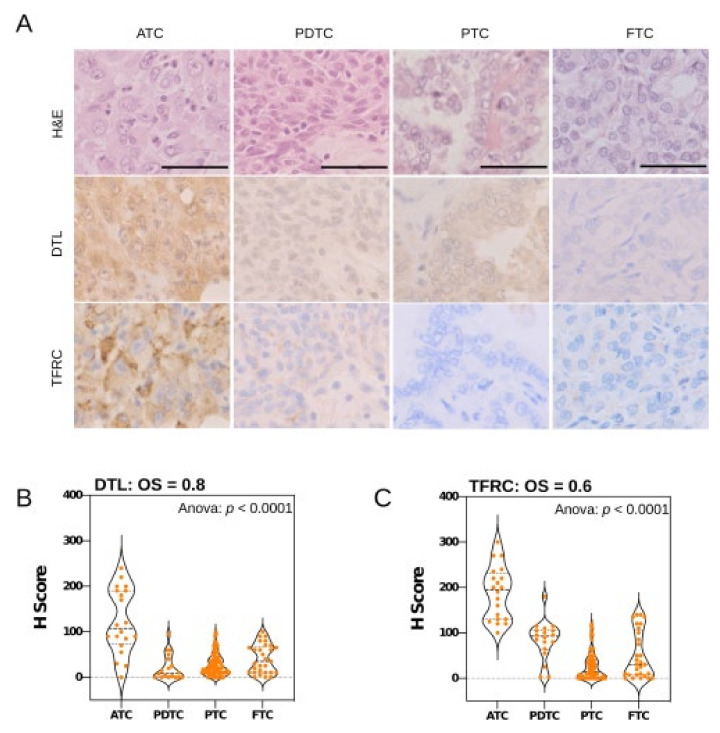
Expression of miRNA-dependent oncogenic factors DTL and TFRC distinguishes ATC. (**A**) Expression of DTL and TFRC analyzed by immunohistochemistry (IHC) in representative samples derived from a tissue microarray (TMA). H&E, hematoxylin eosin staining; magnification 1:40; bars indicate a scale of 50 µm. (**B**,**C**) Evaluation of IHC samples by histoscore (H-score). Violin plots show the H-score distributions determined for DTL (**B**) and TFRC (**C**) in indicated samples. ANOVA testing confirms significantly distinct expression. Gene specific OncoScores (OS) are depicted in top panels.

Aiming to rule out that the analyses are potentially biased by evaluating only miRNA/mRNA data derived from a small and unique cohort, the protein expression of two additional candidate oncogenic factors was investigated in an independent cohort. To this end, the protein expression of DTL (denticleless protein homolog) and TFRC (transferrin receptor), both suggested to be upregulated by miRNA downregulation, was analyzed in an independent thyroid carcinoma tumor cohort (n = 151) by immunohistochemistry. Similar to recent findings reporting the de novo synthesis of IGF2BP1 in ATC [8], these studies confirmed a strong upregulation of both proteins in ATC (Figure 5). Strikingly, the H-scoring of DTL as well as the TFRC protein expression demonstrated a sharp upregulation of both factors in ATC (Figure 5B,C; Table S8). Their upregulation even allowed a clear distinction of ATCs (n = 20) from PDTCs (n = 18), confirming that the presented analysis pipeline is unlikely biased by the small size of the test cohort.

## 4. Discussion

In this study, we provided a comprehensive analysis pipeline to derive disease-relevant miRNA–mRNA target association in ATC. Although the described pipeline settled on a rather small tumor sample cohort and included public data for evaluating the prognostic value of mRNA in thyroid cancer—notably lacking ATC information—as well as essentiality scores determined in ATC-derived cell lines, the presented results appear surprisingly robust, a benefit probably derived from investigating mRNA/miRNA expression by RNA-seq from identical samples side-by-side. Our findings indicate that ATCs are clearly distinguished by a substantial deregulation of miRNA expression and strongly indicate that the severe reorganization of the mRNA transcriptome in ATC is partially driven by miRNA deregulation. Accordingly, the reported findings provide important insights on major gene sets, here only analyzed for well-defined cancer hallmarks, but, more importantly, also indicate key oncogenic as well as tumor-suppressive factors largely regulated by aberrant miRNA expression. For ATC biogenesis, this suggests that the miRNA-dependent control of gene expression is an essential determinant underlying the severe deregulation of the transcriptome observed in ATCs [8].

The miRNome of ATCs is characterized by comparatively few upregulated miRNAs, including key oncomiRs such as miR-146-5p as well as miR-210-3p, which serve various conserved roles in promoting a pro-aggressive and metabolic shift in tumor cell phenotypes [44,45]. However, they are also highly abundant in WDTCs such as the conserved oncomiRs 21-5p, which is even significantly decreased, yet highly abundant in ATC. This suggests that the upregulation of miRNAs has an only modest influence on the reprogramming of the ATC transcriptome and disease progression. Substantially more prominent is a severe downregulation of various miRNAs in ATCs. This has been reported in cancers with mutations in the miRNA processing machinery, specifically in DGCR8 and DICER [20,22]. However, in view of a partial miRNA upregulation and a substantial abundance of some miRNA, e.g., miR-21-5p, the broad downregulation is likely influenced by both, disturbed processing and epigentic/transcriptional deregulation in ATC. Irrespective of the causal association of miRNA downregulation, its reduction is associated with a dominant upregulation and even the de novo synthesis of various mRNAs, as previously reported [8]. In the case of the miR-30/200 families, this downregulation has been linked with a key hallmark of ATC, EMT-like de-/trans-differentiation [17]. The analysis pipeline presented here, however, highlights a pivotal role of miRNA downregulation on the upregulation of various oncogenes or pro-oncogenic factors essential to promoting high proliferation and antagonizing apoptosis. Key examples include the multi-versatile anti-apoptotic survivin (BIRC5) and essential pro-proliferative factor RRM2, which are both actively considered/evaluated as targets for cancer therapy [46,47]. In addition, our studies unraveled the upregulation of major pro-oncogenic drivers in ATC such as the E3-ubiqutin ligases DTL promoting the decay of tumor suppressors such as PDCD4 [48] and the transferrin receptor TFRC (CD71). The latter serves essential roles in promoting a highly proliferative, cancer stem cell-like phenotype [49] and is considered as a therapeutic target in cancer treatment [50]. Its sharp upregulation in ATC may indicate TFRC as a promising avenue for targeted therapy approaches.

Intriguingly, our studies identified the IGF2 mRNA binding protein (IGF2BP1) as a major miRNA-dependent pro-oncogenic factor upregulated in ATC. This supports recent reports identifying IGF2BP1 de novo synthesis as the most robust positive molecular marker of ATC reported so far [8]. In addition, the findings presented here strongly suggest that the de novo synthesis of IGF2BP1 is further promoted by the downregulation of the let-7 family, supporting a pro-oncogenic, let-7 antagonizing role previously demonstrated for IGF2BP1 in other cancer models [11,39]. This provides strong evidence that IGF2BP1 may further enhance miRNA-dependent regulation in ATC, since the main, conserved role of IGF2BP1 in cancer is the enhancement of pro-oncogenic factors by impairing their miRNA-directed inhibition [11,12,13,51]. This suggests a second layer of miRNA-dependent control settling on RNA-binding proteins such as IGF2BP1, which may promote thyroid carcinoma progression by impairing the miRNA-dependent downregulation of oncogenes.

## 5. Conclusions

The presented analyses provide valuable insights and resources to explore the miRNA-dependent regulation of severe transcriptome deregulation in ATC. The MiRNA-dependent deregulation of gene expression identifies a signature comprising 65 genes distinguishing ATC from other thyroid carcinomas of follicular origin.

## Data Availability

The presented data have been deposited in NCBI’s Gene Expression Omnibus and are accessible through GEO Series accession number GSE185719 (https://www.ncbi.nlm.nih.gov/geo/query/acc.cgi?acc=GSE185719, accessed on 19 November 2021). Normalized count data are also available via the R2: Genomics Analysis and Visualization Platform (http://r2.amc.nl, accessed on 19 November 2021; datasets: “Tumor Thyroid Carcinoma—Huettelmaier”) for interactive use and visualization.

## Supplementary Materials

The following are available online at https://www.mdpi.com/article/10.3390/cancers13235913/s1, Figure S1: GSEA of ATCs compared to single WDTC and NT samples, Table S1: Differential expression of mRNAs reanalyzed to HG38, Table S2: Determined miRNA-mRNA interactions ranked by interaction score (IS), Table S3: Hazard ratios from median expression separated TCGA survival data of PTC samples, Table S4: Gene set enrichment analysis of IS ranked target mRNAs, Table S5: Differential expression of miRNAs, Table S6: Parameters of 85 selected miRNAs, Table S7: OncoScore ranked table of top-target mRNAs with best interactions, Table S8: H-score data of tissue microarrays.
