# Supplementary figures and images for: MiRNA Deregulation Distinguishes Anaplastic Thyroid Carcinoma (ATC) and Supports Upregulation of Oncogene Expression
Source: Cancers (Basel). 2021 Nov 24;13(23):5913. doi: 10.3390/cancers13235913 (PMC8657272; doi:10.3390/cancers13235913)

# Hallmark GeneSets

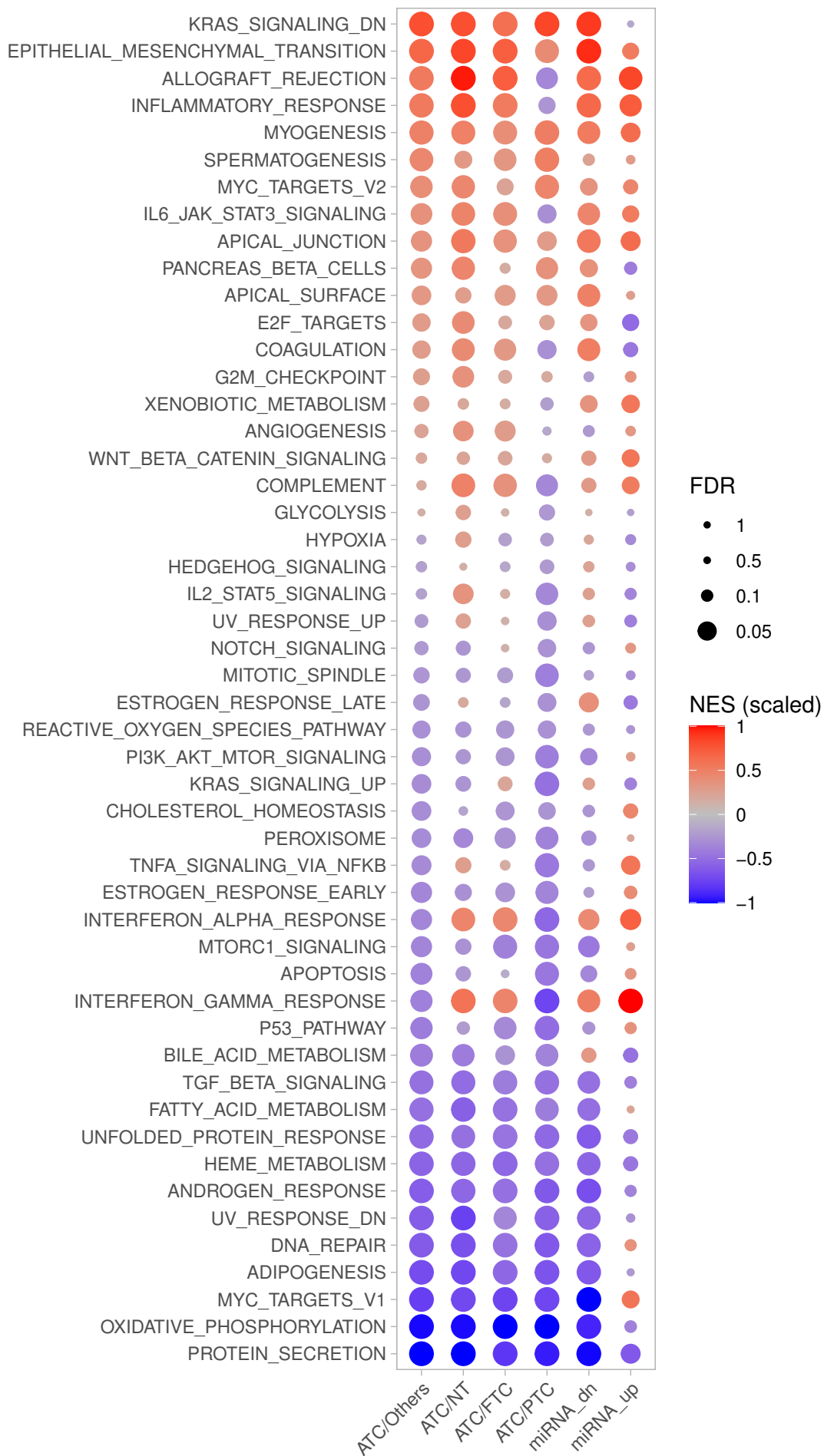

Supplement: Supplementary file 1 [file cancers-13-05913-s001.zip › cancers-1467870-supplementary/Figure_S1.pdf]
